# Effect of Brewery Spent Grain Level and Fermentation Time on the Quality of Bread

**DOI:** 10.1155/2022/8704684

**Published:** 2022-02-23

**Authors:** Tadlo Yitayew, Demewez Moges, Neela Satheesh

**Affiliations:** ^1^Faculty of Chemical and Food Engineering, Bahir Dar Institute of Technology, Bahir Dar University, P. O. Box 26, Bahir Dar, Ethiopia; ^2^Departments of Food Nutrition and Dietetics, Faculty of Agriculture, Sri Sri University, Cuttack, Odisha, India

## Abstract

BSG (brewery spent grain) is the most frequent by-product from the beer industry, which is high in protein, fiber, and minerals. This research was carried out to improve the nutritional content of bread by adding BSG to wheat flour. In this study, five levels (0%, 5%, 10%, 15%, and 20%) of BSG blending ratio and three levels (1, 2, and 3 hrs) of fermentation time were considered. Standard procedures were used to determine the chemical composition of BSG, dough quality, physicochemical composition, and sensory quality of bread. The BSG is composed of 6.19% moisture, 4.01% ash, 8.80% crude fat, 16.80% crude fiber, 21.86% crude protein, 42.30% carbohydrate, 2.57 mg/g calcium, 3.16 mg/g magnesium, and 7.34 mg/g potassium. The dough water absorption (58.53-66.67 ml/100 g), development time (3.43-17.57 min), stability (6.53–12.40 min), and degree of softening (25.33-50.33 FU) were increased significantly (*p* < 0.05) as BSG ratio increased in blending. As the BSG raised, the loaf weight (127.58-148.85 g) was increased and reduced the loaf volume (372.97–366.74 cm^3^). The proximate composition of the BSG blended bread was increased significantly from 33.19 to 45.29% moisture, 1.31 to 3.82% ash, 0.88 to 3.63% crude fat, 0.74 to 8.45% crude fiber, and 8.33 to 14.65% crude protein. The utilizable carbohydrate and energy values were decreased from 53.18 to 34.45% and 2.66 to 2.24 kcal, respectively. The calcium, magnesium, and potassium contents of the bread were increased from 76.44 to 150.93 mg/100 g, 87.12 to 176.81 mg/100 g, and 116.04 to 225.49 mg/100 g, respectively, as the BSG level was increased from 0 to 20%. However, the fermentation time had a significant effect (*p* < 0.05) only on the moisture content, protein content, caloric value, and mineral content of bread. The sensory acceptance of bread was significantly affected (*p* < 0.05) by BSG levels. Finally, by considering the sensory, other functional, and nutritional properties, we concluded that replacing the wheat flour with BSG up to 10% was accepted by the consumers.

## 1. Introduction

Brewery spent grain (BSG) is the main residue left after the separation of wort during the brewing process ([1, 2]; Wolfgang, 2004). Brewery spent grain is the most common brewing by-product (accounting for 85 percent) formed in the beer industry [3, 4]. The BSG produced 31 percent of the original malt weight and 20 kilograms per 100 liters of beer [5]. The annual global production of BSG was accounted for approximately 39 million tons, around 10% produced from Europe [6, 7].

The BSG is composed of barley grain, husk in the largest proportion, a minor fraction of pericarp, and fragments of the endosperm. The chemical composition of BSG varies with barley variety used, malting and mashing conditions, and the quality and type of adjuncts added in the beer production process [8, 9]. Generally, BSG is a lignocellulosic material rich in protein, fiber, and essential amino acids as well as appreciable levels of minerals, polyphenols, vitamins, and lipids [8, 10]. The BSG contains 60-70% of the fiber, predominantly composed of cellulose and hemicelluloses together with xylose, glucose, and arabinose [11]. The BSG is also a good source of valuable proteins (15-26%), including essential amino acids (lysine), representing approximately 30% of the total protein content [12, 13].

Waste disposal in the food industry has become a major issue for maintaining sanitation and preventing contamination of soil, air, and water in recent years. Food industrial waste contains a large concentration of carbohydrates, protein, fats, and minerals and a high value of BOD, COD, and suspended solids that attract insects, rodents, and pathogenic organisms. If BSG is not properly treated, it has a high potential to trigger serious pollution problems [3, 14]. Brewers are currently challenged by keeping the bottom-line costs low and making waste like BSG environmentally friendly by converting it into a value-added commodity due to the current global issue of the green environment [15].

The BSG is a low cost, easily available, and most valuable resource for industrial application [1]. It is an interesting raw material for both food and nonfood applications [16], including animal feed, cultivation of microorganisms, bricks production, compost preparations, and biogas productions, with the manufacture of breakfast cereals, bread, and other baked goods and snacks [4, 17–19]. The brewing process uses approved raw materials for human consumption, so that BSG is a good ingredient for developing new food products [6]. Incorporating BSG in the formulation of bakery products (bread, extrudate, and snacks) has health promoting effects, including lowering the risk of cardiovascular diseases, constipation, colon cancer, obesity, and diabetes [6, 20].

Brewing is one of the fastest growing industries in Ethiopia. The utilization of BSG is limited to animal feed, but no attention is paid to its utilization for food and nonfood applications. Since the brewing process uses materials approved for human consumption and BSG is a good source of dietary fiber, protein, fat, vitamins, and minerals, partial incorporation of BSG in formulated functional foods (biscuits, bread, cookies, and extrudates) may provide different benefits to human health [12, 20, 21].

Bread from wheat flour is the most common and widely consumed food by all age groups. However, bread prepared from wheat flour is considered nutritionally poor. The partial replacement of wheat flour with gluten free formulations improves the nutritional quality of bakery products and satisfies consumers' demands for healthy foods [22]. Hence, the main objective of this study is to investigate the rheological property of BSG supplemented dough, the effect of different blending levels of BSG, and fermentation time on the physical properties, proximate composition, mineral composition, and sensory quality of bread.

## 2. Materials and Methods

### 2.1. Collection of Raw Materials and Processing

Fresh BSG was collected from Dashen Brewery, Gondar, Ethiopia, while wheat flour (First class, Merkeb Union Flour Factory), Instant Bakery Yeast (Saf-Instant, Turkey), and table salt (Iodized salt, Walia Salt Factory) were purchased from the local market (Bahir Dar city, Ethiopia). The research is conducted at the Food Technology laboratories in the Faculty of Chemical and Food Engineering, Bahir Dar Institute of Technology, Bahir Dar University. The water in the BSG was removed by manual hand pressing, and the remaining moisture was removed by in-house air drying at room temperature. After drying, the dried BSG was milled by local cereal mills and sieved by 355 *μ*m sieve size (Fritsch, Germany).

### 2.2. Experimental Design

A two-factor factorial design arranged in completely randomized design (CRD) was used in this study. The blending ratio was considered in five levels (0%, 5%, 10%, 15%, and 20%) and fermentation time in three levels (1, 2, and 3 hr). The entire experiment was conducted in triplicate, and the total runs were 45.

### 2.3. Dough Preparation and Bread Making

The dough was prepared by adding water as per the water absorption value obtained from the farinograph results (60-75 ml/100 g); 2% table salt and 1% yeast (dry ingredients) were added to the bread formulation. A laboratory scale dough mixer (HS 130 Spiral Mixer, China) was used to mix the ingredients and dough development. The resulting dough was allowed to ferment for a specified time as designed. The fermented dough was molded by hand and placed into pregreased baking trays. The baking was performed at 230°C for 30 min. The baked loaves were carefully removed from the pans and allowed to cool. Furthermore, the samples were immediately used to determine proximate, mineral, and sensory analysis.

### 2.4. Analysis of Dough Properties

The water absorption and mixing characteristics of the composite dough samples were done using the Farinograph-E instrument (Farinograph, China) according to AACC [23]. The flour sample of 300 g on a 14% moisture basis was weighed and taken into a farinograph mixing bowl. Water from a burette at 30°C was added to the flour and mixed to form the dough. Furthermore, the farinograph was developed using the standard procedures. Water absorption, dough development time, dough stability, and degree of softening were recorded from the farinograph curves.

### 2.5. Determination of Bread Loaf Physical Properties

Loaf weight was determined by using an electronic digital balance with an accuracy of 0.001 g. The loaf volume of bread was measured using the grain displacement method as reported by Makinde and Akinoso [24] with minor modification in dimension of the measuring box. A box of fixed dimensions (22.73 × 15.27 × 16.21 cm) with an internal volume of 5626.28 cm^3^ was filled with sorghum grains, and the total weight was noted (*C*). The preweighed loaf (*W*) was placed in the box, and seeds of known weight were used to fill the box to the top level. The loaf volume was determined by equations (1), (2), and (3).

(1) $$\begin{array}{c} \text{Seed Displaced by Loaf }\left(L\right)=W+\text{Over spill weight}-C, \end{array}$$

(2) $$\begin{array}{c} V=\frac{L*5626.28^{3}}{C}, \end{array}$$

where *V* is the loaf volume, *L* is the seed displaced by loaf, *C* is the sorghum weight, and 5626.28 cm^3^ is the internal volume of measuring device.

Loaf specific volume was obtained by dividing the loaf volume by its corresponding weight

(3) $$\begin{array}{c} \text{Loaf Specific Volume}=\frac{\text{Loaf Volume }\left({\text{cm}}^{3}\right)}{\text{Loaf Weight }\left(\text{g}\right)}. \end{array}$$

### 2.6. Proximate Analysis of BSG Flour and Bread

Proximate analysis of BSG flour and bread samples was carried out according to the AOAC [25] procedure. The moisture content was determined gravimetrically by using hot air oven method number 925.09. The ash was determined by using muffle furnace according to the AOAC method number 923.03, crude fat was determined by using Soxhlet extraction method ([25], Method: 923.03), crude fiber was determined by using nonenzymatic gravimetric method of AOAC method number 920.39, and Kjeldahl method was used for crude protein analysis according to the standard AOAC [25] method number 962.09, respectively. The total percentage of utilizable carbohydrate content was determined by the difference method. The gross energy was calculated according to equation (4).

(4) $$\begin{array}{c} \text{Energy},\text{kcal}=\left(\text{Crude Fat}*9\right)+\left(\text{Crude Protein}*4\right)+\left(\text{Utilizable carbohydrate}*4\right). \end{array}$$

### 2.7. Mineral Analysis

The concentrations of calcium and magnesium in bread samples were measured by an atomic absorption spectrophotometer (Perkin-Elmer, Model 3100, USA) (Gimblet et al., 1966) after wet digestion of 1 g sample [26]. From the resulting sample solution, 10 ml was taken in to 50 ml conical flask and mixed with 4 ml of concentrated nitric acid and diluted with deionized water up to the calibration mark. From the resulting sample solution, 10 ml was measured in a 50 ml conical flask and mixed with 2 ml of molybdate solution, 4 ml of concentrated nitric acid, and 4 ml hydrazine sulphate and heated in a water bath at 60°C until a blue color was developed. A standard stock solution of calcium, magnesium, and potassium was prepared by appropriate dilution of stranded pure metals. The absorbance of calcium, magnesium, and potassium was measured at 422.7 nm, 285.2 nm, and 830 nm wavelengths, respectively. The concentration of the sample solution is obtained according to the standard curve. The concentration of the phosphorus content was determined by UV-vis spectrophotometer (Cary 60 UV–Vis, Malaysia) after wet digestion of 1 g of sample according to the procedure of Ganesh et al. [27].

### 2.8. Sensory Evaluation

The prepared breads were coded randomly with three digits. The samples are provided to the untrained panelists for sensory evaluation. The panelists were asked to give the sensory scoring by considering the color, flavor, taste, crumb texture, and overall acceptability of the bread using a nine-point hedonic scale according to the method reported by Ikuomola et al. [28].

### 2.9. Data Analysis

The Analysis of Variance (ANOVA) was conducted to determine the difference between the treatments by using the Statistical Analysis System (SAS) software (9.1.3) with the general linear model. The mean comparison was done by Duncan multiple range test, and significance was considered at *p* ≤ 0.05.

## 3. Results and Discussion

### 3.1. Proximate and Mineral Composition of BSG

The results on proximate composition and mineral composition are presented in Table 1. As seen from the table, BSG is a good source of fat, fiber, and protein with minerals in trace amounts. The ash content was 4.01%, the crude fat content was 8.79%, the crude fiber content was 16.95%, and the crude protein content was 21.86% with 2.50 mg/g calcium, 3.10 mg/g magnesium, and 7.40 mg/g potassium content. Different studies are [9, 13, 29] conducted on brewery spent grain and concluded that BSG is a good source of protein, fat, ash, and dietary fiber. There was a slight variation in the composition of the crude protein, crude fat, crude fiber, and ash content of the BSG in different reported studies. This variation may be attributed to the barley variety, maturity of the barley used in the process, malting and mashing conditions, quality, and type of adjuncts added [9]. In general, protein and fibers are dominantly available in BSG. The carbohydrate content of BSG is lower than that of barley due to the extensive amylolysis process of mashing. The BSG is also a good source of trace metals. The reports of Madubuike and Okolo [30]; Waters et al. [13] showed that BSG had a high concentration of calcium, magnesium, and phosphorus and trace amounts of zinc and iron.

### 3.2. The Farinograph Property of Dough

Table 2 depicts the effect of BSG level on the water absorption, dough development time, dough stability, and degree of softening.

The water absorption was increased significantly (*p* < 0.05) from 58.40 to 66.67 ml/100 g as the BSG level was increased from 0 to 20%. An increase in the water absorption of the BSG blended wheat flour was attributed to the presence of high protein and nonstarchy polysaccharides (fibers) in the BSG. The work of Steinmacher et al. [31] and Stojceska and Ainsworth [6] reported that the water absorption increases from 58 to 61% with 10% BSG addition and 63.70-72.30% with 12% BSG addition, respectively. The higher fiber content of BSG and high protein content of BSG provide high water absorption and consequently provide good baking performance [10, 32].

Similarly, the dough development time was also increased from 3.43 to 17.57 min as the BSG level increased from 0 to 20%. This may be attributed to the high fiber and protein content of BSG, prolonging the dough development time. However, the dough stability and degree of softening were not increased linearly. The dough stability and degree of softening were initially increased from 6.53 to 12.40 min and 25.33 to 50.33 FU, respectively. In contrast, the dough stability and degree of softening were decreased to 9.90 min and 45.33 FU by the addition of 15% BSG, and the result was not detected at 20% BSG level. These trends in dough stability and degree of softening are attributed to the long mixing time and high shear force applied in addition to the high fiber and protein content. Steinmacher et al. [31] reported that dough made with composite flours had longer development time and lower stability compared to wheat flour dough. The study of Stojceska and Ainsworth [6] also reported an increase in dough development time and dough stability and a decrease in the degree of softening when wheat flour is blended with BSG.

The dough development time depends on the water absorption speed of flour constituents to form a smooth and homogenous appearance. At the same time, strong wheat has higher water absorption, good extensibility, longer dough development time, and stability [10]. Longer development time indicates that the dough was developed slowly when additional fiber and protein were present in the flour. The decrease in dough stability and degree of softening may be attributed to the high interference between the protein and fiber content in dough. Generally, the addition of high fiber content raw materials may affect the dough properties significantly [33].

### 3.3. The Physical Properties of Bread

The ANOVA showed that BSG had a significant effect (*p* < 0.05) on the loaf weight, loaf volume, and specific volume of bread loaf as presented in Table 3. The loaf weight was increased from 127.58 to 148.85 g as the BSG level was increased from 0 to 20%. This incensement in loaf weight was attributed to the high fiber and protein content in the BSG. As the BSG level increased in the dough, the water absorption capacity increased, and it leads to the reduction in the carbon dioxide holding capacity of the dough. The works of Mongi et al. [34] and Mudau et al. [35] also reported the increase in bread loaf weight by the addition of high fibrous ingredients in the dough.

The loaf volume and specific loaf volume of bread were decreased from 372.97 to 366.74 cm^3^/g and 2.89 to 2.46 cm^3^/g, respectively, as the BSG level was increased from 0 to 20%. This trend may be attributed to the interaction between fiber and protein and leads to weakening of the gluten network of the dough and reduces the gas holding capacity of the dough. Amoriello et al. [36] and Ktenioudaki et al. [37] showed that the specific volume of bread loaves was decreased as the BSG addition in bakery product formulation. The high fiber content of BSG reduces product volume and elasticity during the dough preparation [33, 38].

In this study, fermentation time had a nonsignificant effect (*p* < 0.05) on the weight, volume, and specific volume of bread loaves. This was attributed to the presence of high fiber content in the BSG, and this fiber enhances the water absorption of the dough and weakens the gluten network and gas holding capacity of the bread loaves. In contrast, Aplevicz et al. [39] reported that as the fermentation time was increased, the loaf volume of bread also increased significantly.

### 3.4. The Proximate Composition of Bread

The results in Table 4 showed the effect of BSG level and fermentation time on the nutritional quality of bread. The level of BSG addition had a significant effect (*p* < 0.05), while fermentation time had a nonsignificant effect (*p* > 0.05) on the bread proximate composition.

The moisture content of bread was significantly affected (*p* < 0.05) by the BSG level, and it was increased from 33.19 to 45.29% as the BSG level increased from 0 to 20%. This may be attributed to the presence of high fiber content of BSG, the baking temperature, and time. The fiber absorbs higher quantities of water during mixing and leading to high water retention capacity. Similarly, the work of Fărcaş et al. [12] showed that the moisture content of the bread was reported higher as the BSG level was increased. The baking temperature and time affect the starch gelatinization and which in turn affect the moisture content of the bread [40]. The fermentation time significantly (*p* < 0.05) reduced the moisture content of bread as the fermentation time was increased from 1 to 3 hr. This decreasing trend in moisture content of bread may be attributed to the evaporation of water from the dough surface during the extended fermentation duration. The work of Siddiqi et al. [41] also showed that the moisture content of the bread was decreased as the fermentation time was increased.

The BSG level also had a significant effect (*p* < 0.05) on the ash content of bread. It increased from 1.19 to 3.82% as the BSG level was increased from 0 to 20%. This may be attributed to the presence of higher amounts of minerals in the cereal grains. Musa et al. [42] indicated the addition of bran increases the ash content of the bread. Similarly, whole grain bakery products are reported to be rich in ash content.

The crude fat content of bread was significantly (*p* < 0.05) increased from 0.88 to 3.83% as the BSG level was increased from 0 to 20%. This may be due to the BSG contains part of the barley germ, and usually, the cereal germ is rich in fat content. The work of Fărcaş et al. [12] and Ktenioudaki et al. [37] also showed that the fat content of the baked products is increased as the BSG level increased.

The bread crude fiber and protein contents were increased from 0.74 to 8.45% and 8.33 to 14.45%, respectively, as the BSG level increased from 0 to 20%. This may be attributed to the brewing process is focusing more on starch hydrolysis and little on proteolysis; thus, BSG is a good source of fiber and protein. Previous studies [5, 30, 43] also showed that BSG is rich in fiber and proteins. The study of Fărcaş et al. [12] and Stojceska and Ainsworth [6] showed that BSG addition increased the crude fiber and protein content of the bakery product.

The fermentation time also had a significant effect (*p* < 0.05) on the protein content of the bread. It was increased from 11.95 to 12.71% as the fermentation time was increased from 1 to 3 hr. This increment may be attributed to the loss of dry matter during fermentation and degradation of complex proteins by microbial action, and proteins may be produced as a by-product of the fermentation process. Similerly, Day and Morawicki [44] and Nkhata et al. [45] also reported an increment of protein continents as fermentation time extends in fermented foods.

The utilizable carbohydrate and calorific value of bread were decreased from 53.93 to 35.07% and 2.60 to 2.28 kcal, respectively, as the BSG composition increased. This trend may be attributed to the mashing operation during the brewing process which is aimed to hydrolysis of starch into fermentable sugar [13], which reduces the carbohydrate content in the BSG. The calorific value is the sum of crude fat, protein, and utilizable carbohydrate content with their multiplication factor. Thus, the caloric value also reduces accordance with the carbohydrate content.

### 3.5. The Mineral Content of Bread

As shown in Table 5, both the BSG level and fermentation time had a significant effect (*p* < 0.05) on the mineral content of the bread. The calcium, magnesium, and potassium contents of the bread were increased from 76.44 to 150.93 mg/100 g, 87.12 to 176.81 mg/100 g, and 116.04 to 225.49 mg/100 g, respectively, as the BSG level was increased from 0 to 20%. This may be attributed to that BSG levels as it is a good source of minerals.

The BSG is mainly composed of the husk of grain and minerals in cereals present in the outer layer of their husk. The reports of Ikram et al. [46] and Waters et al. [13] also confirmed that BSG is rich in calcium, magnesium, potassium, and other minerals in trace amount. The study of Fărcaş et al. [12] showed an increased content of mineral from 0.44% to 1.29% for bread with 20% BSG addition. The work of Sharif et al. [47] also indicates that the addition of defatted rice bran can also increase the mineral content of cookies significantly. In addition, Tizazu et al. [48] also showed that germination or preprocessing of cereal grains could reduce the phytate level and increase the mineral bioavailability.

Similarly, the fermentation time also had a significant effect (*p* < 0.05) on the mineral content of the bread. The calcium, magnesium, and potassium contents of the bread were increased from 110.37 to 119.55 mg/100 g, 120.35 to 128.59 mg/100 g, and 160.81 to 172.43 mg/100 g, respectively, as the fermentation time was increased from 1 to 3 hr. This may be attributed to the fact that fermentation of food products improves the nutritional quality as well as the bioavailability of minerals by the breakdown of oxalates and phytates. The work of Buta and Emire [49] showed that fermentation had a significant effect on the mineral content of soybean blends of weaning food. On the other hand, the work of Ojokoh et al. [50] on the breadfruit and cowpea blend flours showed that fermentation reduces the oxalate and phytate content. The work of Assohoun et al. [51] also showed that fermentation improved the mineral content (Ca, Mg, P, Z, Fe, etc.) of fermented foods as the phytate level decreased and loss of dry matter as the fermenting yeast utilizes the carbohydrate and protein.

### 3.6. Sensory Acceptability of BSG Incorporated Bread

As showed in Table 6, the sensory acceptance of the bread prepared from wheat and BSG flour at different levels was decreased significantly (*p* < 0.05) as the BSG level was increased from 0 to 20%. The panelist score for bread color was decreased significantly (*p* < 0.05) from 7.58 to 5.58. The color of the bread appears darker as the BSG flour substitution level was increased from 0 to 20%. This may be due to the dark brown color of BSG flour and the high fiber content of BSG. The study conducted by Lukinac et al. [52] and Petrović et al. (2015) on bread and cookies also reported that the color of the product turns darker as the substitution level of BSG flour was increased as they measured the color of the product instrumentally.

The taste and flavor of the bread were also negatively affected (*p* < 0.05) by the addition of higher levels of BSG. The panelists were given the sensory score of 7.63-6.05 and 8.00-6.05 for taste and flavor, respectively, out of nine-point hedonic scale. This trend may be attributed to the BSG; it is a poor source of sugar as the sugar is extracted, and cooked malt flavor is also developed during the mashing process. The study of Ikuomola et al. [28] showed that panelist score for taste and flavor was decreased as the level of malted barley increased in the baked foods.

The crumb texture was also significantly affected (*p* < 0.05) by the BSG level. The panelist score was decreased from 7.95 to 5.58 as the BSG level was increased from 0 to 20%. The crumb texture appeared rough, harder, and cracked. This may be attributed to the presence of high fiber, and protein absorbs much water causing harder bread structure. The addition of fiber-rich ingredients increases the hardness of the product (Petrović et al., 2015; [6]). The overall acceptability of bread was decreased significantly (*p* < 0.05) from 7.74 to 6.05 as the BSG level was increased from 0 to 20%. This may be attributed to the cumulative effect of color, taste, flavor, and crumb texture. Similarly, different scientists [12, 13, 53, 54] reported that the addition of BSG significantly alters the sensory acceptance of different baked products.

## 4. Conclusion

The composite bread from BSG and wheat flours was developed and analyzed to assess the effect of BSG level and fermentation time on the nutritional value of bread. The BSG is nutrient rich, and its utilization in food application will enhance the economic potential of the brew house as well as improving the nutritional value of bread. The BSG level significantly affects (*p* < 0.05) the dough properties, the proximate composition, mineral content, and sensory acceptance of bread. The ash, crude fat, crude fiber, and crude protein of the bread were increased from 1.31 to 3.82%, 0.88 to 3.63%, 0.74 to 8.45%, and 8.33 to 14.65%, respectively, as the BSG level increased from 0 to 20%. Similarly, the calcium, magnesium, and potassium content of the bread was increased from 76.44 to 150.93 mg/100 g, 87.12 to 176.81 mg/100 g, and 116.04 to 225.49 mg/100 g, respectively, as the BSG level was increased from 0 to 20%, while the carbohydrate content and the sensory acceptance were significantly affected by the BSG level. The fermentation time also significantly increased the protein, calcium, magnesium, and potassium content of the bread from 11.95 to 12.71%, 110.37 to 119.55 mg/100 g, 120.35 to 128.59 mg/100 g, and 160.81 to 172.43 mg/100 g, respectively, as the fermentation time was increased from 1 to 3 hr. BSG is a rich source of protein and fiber, and incorporating BSG flour in bakery products has a health promoting effect. However, the addition of high amount of BSG flour negatively affects the physical properties and sensory acceptance of bread. We can conclude from this study that the optimum BSG flour level in bread formulation never exceeds 10%.

## Figures and Tables

**Table 1:** Proximate and mineral composition (% dry matter) of brewery spent grain.

Physicochemical composition	Values (%)
Moisture content	6.09 ± 0.77
Ash	4.01 ± 0.25
Crude fat	8.79 ± 0.33
Crude fiber	16.95 ± 0.45
Crude protein	21.86 ± 0.31
Carbohydrate	42.30 ± 0.47
Mineral composition	Values (mg/g)
Calcium	2.57 ± 0.01
Magnesium	3.16 ± 0.02
Phosphorus	7.34 ± 0.02

Values are presented as mean ± standard deviation of triplication.

**Table 2:** Farinograph properties (water absorption, dough development time, dough stability, degree of softening) of dough prepared from the blend of wheat and BSG flours.

BSG level, %	Water absorption (ml/100 g)	Dough development time (min)	Dough stability (min)	Degree of softening (FU)
0	58.40 ± 1.16a	3.43 ± 0.21a	6.53 ± 0.15a	25.33 ± 2.51a
5	59.53 ± 1.25a	4.60 ± 0.20b	11.60 ± 0.10c	36.00 ± 2.64a
10	62.23 ± 1.09b	6.96 ± 0.58c	12.40 ± 0.91c	50.33 ± 4.72c
15	65.18 ± 1.48c	8.93 ± 1.00d	9.90 ± 0.70b	45.33 ± 2.52c
20	66.67 ± 1.13c	17.57 ± 0.40e	ND	ND
*p* values (BSG level^∗^ fermentation durations)	<0.0001	<0.0001	<0.0001	<0.0001

The values are mean ± standard deviation of three observations. ND: not determined. Means followed by different letters within the column show statistically significant difference (*p* < 0.05).

**Table 3:** Physical properties of the bread prepared with different levels of BSG and fermentation durations.

BSG level (%)	Fermentation time (hr)	Loaf weight (g)	Loaf volume (cm^3^)	Specific loaf volume (cm^3^/g)
0	1	129.31 ± 1.23a	370.61 ± 4.45abcd	2.87 ± 0.05ef
	2	129.12 ± 2.99a	372.85 ± 2.09bcd	2.89 ± 0.07f
	3	127.58 ± 3.32a	372.97 ± 3.89bcd	2.92 ± 0.08f
5	1	135.89 ± 1.52b	368.86 ± 3.69ab	2.71 ± 0.03d
	2	133.74 ± 1.81b	373.45 ± 1.30ab	2.79 ± 0.04e
	3	133.60 ± 1.38b	374.57 ± 1.41d	2.80 ± 0.03e
10	1	142.64 ± 2.72c	369.48 ± 1.96abc	2.59 ± 0.05bc
	2	142.44 ± 3.41cde	369.82 ± 1.27abc	2.60 ± 0.07bc
	3	141.84 ± 2.52cd	371.16 ± 0.97abcd	2.62 ± 0.04c
15	1	146.38 ± 1.67ef	368.58 ± 0.88a	2.51 ± 0.03a
	2	145.83 ± 1.60cdef	368.62 ± 0.3.17a	2.53 ± 0.03ab
	3	145.24 ± 0.48def	371.07 ± 0.06abcd	2.56 ± 0.13dab
20	1	148.85 ± 1.24f	366.74 ± 0.45a	2.46 ± 0.36a
	2	148.82 ± 0.63f	368.23 ± 0.53ab	2.47 ± 0.84a
	3	148.14 ± 1.63f	369.33 ± 2.42abc	2.49 ± 1.48a
*p* values (BSG level^∗^ fermentation durations)		<0.0001	0.0090	<0.0001

The values are mean ± standard deviation of three observations. Means followed by different letters within the column show statistically significant difference (*p* < 0.05).

**Table 4:** The effect of BSG and fermentation on bread physicochemical and physical properties.

BSG level, %	Fermentation time, hr	Moisture, %	Ash, %	Crude fat, %	Crude fiber, %	Crude protein, %	Utilizable CHO, %	Caloric value, kcal
0	1	36.23 ± 2.05de	1.33 ± 0.35a	0.93 ± 0.41cd	0.86 ± 0.12a	8.33 ± 0.12a	53.18 ± 2.72e	2.54 ± 0.07ef
	2	35.55 ± 0.92d	1.19 ± 0.50a	0.99 ± 0.56cd	0.74 ± 0.29a	8.99 ± 0.21b	53.28 ± 0.90e	2.58 ± 0.04f
	3	33.19 ± 2.43c	1.42 ± 0.18a	0.88 ± 0.22c	0.80 ± 0.39a	9.47 ± 0.40c	55.04 ± 2.17e	2.66 ± 0.10f
5	1	39.31 ± 0.83fg	2.69 ± 0.91b	1.29 ± 0.61cde	1.53 ± 0.23a	10.76 ± 0.33d	45.96 ± 1.11d	2.39 ± 0.09d
	2	38.45 ± 0.50f	2.94 ± 0.44b	1.73 ± 0.43def	1.43 ± 0.10a	11.42 ± 0.16e	44.46 ± 1.26d	2.43 ± 0.03ed
	3	37.91 ± 1.67ef	3.13 ± 0.80b	1.99 ± 0.50defg	1.51 ± 0.13a	11.87 ± 0.17e	45.11 ± 2.05d	2.46 ± 0.12ed
10	1	41.90 ± 0.78h	3.82 ± 0.04b	2.40 ± 1.04fghi	4.91 ± 1.68b	13.11 ± 0.29f	38.76 ± 0.45c	2.29 ± 0.08bc
	2	41.77 ± 1.67h	3.17 ± 0.77b	2.15 ± 1.25efgh	4.51 ± 1.56b	13.43 ± 0.12fg	39.48 ± 3.01c	2.31 ± 0.03c
	3	41.41 ± 0.68gh	3.09 ± 0.47b	2.38 ± 0.36fghi	5.24 ± 1.14b	13.54 ± 0.37fgh	39.17 ± 0.30c	2.32 ± 0.02c
15	1	42.92 ± 0.82hi	3.01 ± 0.48b	2.90 ± 0.20ghij	5.28 ± 2.43b	13.61 ± 0.32gh	37.57 ± 1.45bc	2.31 ± 0.03bc
	2	42.83 ± 0.49hi	3.04 ± 0.83b	2.56 ± 0.53ghij	4.82 ± 0.99b	13.73 ± 0.25gh	37.84 ± 1.45bc	2.29 ± 0.06bc
	3	42.13 ± 1.30hji	3.18 ± 1.26b	3.00 ± 024ghij	7.15 ± 1.07c	14.03 ± 0.24hi	36.67 ± 2.01ab	2.30 ± 0.09abc
20	1	45.29 ± 0.73j	2.83 ± 0.75b	3.22 ± 0.31hij	7.86 ± 0.53c	13.95 ± 0.12hi	34.71 ± 1.41a	2.24 ± 0.04a
	2	44.80 ± 0.81ij	2.70 ± 0.57b	3.63 ± 0.39j	8.45 ± 1.22c	14.41 ± 0.41ij	34.45 ± 1.16a	2.28 ± 0.03ab
	3	43.30 ± 1.24hij	2.67 ± 0.35b	3.33 ± 0.52ij	8.38 ± 0.35c	14.65 ± 0.31j	36.04 ± 0.54a	2.33 ± 0.06abc
*p* value	BSG level	<0.0001	<0.0001	<0.0001	<0.0001	<0.0001	<0.0001	<0.0001
	Fermentation	0.0173	0.7178	0.6985	0.2214	<0.0001	0.7956	0.0419
	BSG level^∗^ fermentation durations	<0.0001	0.0004	<0.0001	<0.0001	<0.0001	<0.0001	<0.0001

The values are mean ± standard deviation of three observations. Means followed by different letters within the column show statistically significant difference (*p* < 0.05).

**Table 5:** The mineral content (calcium, magnesium, phosphorus) of BSG incorporated bread.

BSG level (%)	Fermentation time (hr)	Calcium, mg/100 g	Magnesium, mg/100 g	Phosphorus, mg/100 g
0	1	76.44 ± 2.55a	87.12 ± 1.12a	116.04 ± 2.45a
	2	79.74 ± 0.55ab	91.05 ± 0.54ab	120.86 ± 0.01b
	3	81.07 ± 1.55b	93.00 ± 1.25b	123.04 ± 0.21b
5	1	90.11 ± 1.47c	102.07 ± 0.13c	135.12 ± 0.89c
	2	95.90 ± 1.90d	103.82 ± 0.58c	140.15 ± 0.88d
	3	103.77 ± 2.04e	104.30 ± 0.46c	145.71 ± 1.67e
10	1	111.94 ± 2.14f	110.34 ± 1.64d	155.19 ± 2.52f
	2	118.41 ± 1.03g	115.93 ± 2.25e	163.23 ± 2.19g
	3	122.74 ± 1.87h	117.69 ± 1.11e	167.29 ± 1.98g
15	1	130.31 ± 1.25i	140.91 ± 3.88f	187.81 ± 3.42h
	2	133.00 ± 1.72i	145.83 ± 1.93g	192.88 ± 2.44i
	3	139.26 ± 0.45j	151.18 ± 2.07h	200.62 ± 1.68j
20	1	143.07 ± 0.96k	161.30 ± 2.82i	209.91 ± 1.25k
	2	147.02 ± 0.27l	170.47 ± 2.96i	218.66 ± 1.80l
	3	150.93 ± 2.42m	176.81 ± 2.07k	225.49 ± 3.00m
*p* value	BSG level	<0.0001	<0.0001	<0.0001
	Fermentation time	<0.0001	<0.0001	<0.0001
	BSG level^∗^ fermentation durations	<.0001	<.0001	<.0001

The values are mean ± standard deviation of three observations. Means followed by different letters within the column show statistically significant difference (*p* < 0.05).

**Table 6:** Sensory evaluation results of bread prepared by addition of BSG on 9-point hedonic scale.

BSG level (%)	Fermentation time (hr)	Color	Taste	Flavor	Crumb texture	Overall acceptance
0	1	7.37 ± 0.60c	7.63 ± 0.49b	7.53 ± 0.61b	7.47 ± 0.61de	7.42 ± 0.51a
	2	7.58 ± 0.51c	7.53 ± 0.61b	7.68 ± 0.58b	7.95 ± 0.40e	7.53 ± 0.51a
	3	7.47 ± 0.51c	7.63 ± 0.49b	8.00 ± 0.47b	7.73 ± 0.45de	7.74 ± 0.45b
5	1	7.26 ± 0.99c	7.42 ± 0.84b	7.53 ± 0.90b	7.53 ± 1.02de	7.63 ± 0.83b
	2	7.32 ± 1.00c	7.16 ± 0.69b	7.58 ± 0.77b	7.37 ± 0.90d	7.53 ± 0.61b
	3	7.21 ± 0.71c	7.26 ± 0.87b	7.63 ± 0.89b	7.37 ± 0.68d	7.32 ± 0.67b
10	1	7.05 ± 0.62c	7.63 ± 0.76b	7.63 ± 0.96b	7.47 ± 1.02de	7.53 ± 0.84b
	2	7.05 ± 0.40c	7.53 ± 0.61b	7.74 ± 0.73b	7.42 ± 0.90de	7.74 ± 0.56b
	3	7.16 ± 0.69c	7.37 ± 0.83b	7.93 ± 0.71b	7.42 ± 0.77de	7.47 ± 0.51b
15	1	5.95 ± 0.78b	6.32 ± 0.67a	6.21 ± 0.92a	6.42 ± 0.69c	6.42 ± 0.61a
	2	6.21 ± 0.79b	6.11 ± 0.57a	6.58 ± 0.61a	6.21 ± 0.79b	6.05 ± 0.52a
	3	5.58 ± 0.84a	6.26 ± 0.73a	6.05 ± 0.85a	6.26 ± 0.56bc	6.26 ± 0.73a
20	1	5.84 ± 0.69ab	6.11 ± 0.74a	6.11 ± 0.86a	5.68 ± 0.67a	6.21 ± 0.63a
	2	5.58 ± 0.69a	5.95 ± 0.52a	6.05 ± 0.78a	5.84 ± 0.69ab	6.16 ± 0.60a
	3	6.26 ± 0.65b	6.05 ± 0.62a	6.16 ± 0.51a	6.21 ± 0.71b	6.37 ± 0.59a
*p* value	BSG level^∗^ fermentation durations	<0.0001	<0.0001	<0.0001	<0.0001	<0.0001

The values are presented in mean ± standard deviation of 50 observations. Means followed by different letters within the column show statistically significant difference (*p* < 0.05).

## Data Availability

All the data is included in the manuscript.
